# The Rundown of Dietary Supplements and Their Effects on Inflammatory Bowel Disease—A Review

**DOI:** 10.3390/nu12051423

**Published:** 2020-05-14

**Authors:** Bartosz Malinowski, Michał Wiciński, Maya M. Sokołowska, Nicholas A. Hill, Monika Szambelan

**Affiliations:** Department of Pharmacology and Therapeutics, Faculty of Medicine, Collegium Medicum in Bydgoszcz, Nicolaus Copernicus University, M. Curie 9, 85-090 Bydgoszcz, Poland; wicinski4@wp.pl (M.W.); msokolowska@trentu.ca (M.M.S.); originnikko91@gmail.com (N.A.H.); moniassz410@gmail.com (M.S.)

**Keywords:** inflammatory bowel disease, Crohn’s disease, ulcerative colitis, diarrhea, constipation, abdominal cramping/pain, fibers, probiotics, polyphenols, fatty acids, low FODMAP

## Abstract

Inflammatory bowel diseases, including Crohn’s disease and ulcerative colitis, are a life-long, chronic, and relapsing problem affecting 11.2 million people worldwide. To date, there is pharmacological therapy to treat symptoms such as diarrhea, constipation, and abdominal cramping/pain. These medications also help to alleviate everyday discomfort; however, there are no curative therapies. Recent studies have investigated the combination of pharmacological treatment along with nutritional interventions to improve quality of life and risk of disease relapse. Dietary supplements, specifically probiotics, polyphenols, fibers, fatty acids and low fermentable oligosaccharide, disaccharide, monosaccharide, and polyol diets (FODMAP diets), have been closely looked at to determine their effect, if any, on the development of inflammatory bowel disease and its course of progression. Approximately 30 studies were carefully reviewed and analyzed to appreciate the value of these above-mentioned supplements and their influence on this gastrointestinal disease. After analysis, it has been demonstrated that by implementing fibers, polyphenols, and fatty acids, as well as keeping a low-saccharide diet for those patients with Crohn’s disease and ulcerative colitis can improve quality of life and invoke clinical remission. Some polyphenols, specifically curcumin and resveratrol, have proved to decrease disease activity in studies reviewed. Although these studies have become a topic of recent interest, it would be of great value to doctors and patients alike, to continue in this direction of research and to improve the findings for best treatment substances and dosages. This would lead to increased quality of life and disease control leading to fewer complications in the future.

## 1. Introduction

Inflammatory bowel disease (IBD) is a life-long problem around the world. It has been defined by the Center of Disease Control (CDC) as chronic inflammation of the gastrointestinal tract, and includes Crohn’s disease and ulcerative colitis [1]. It has been estimated that IBD affects around 11.2 million people worldwide, with a higher prevalence in Europe and North America [2]. IBD has been known to affect women slightly more than men, and usually those aged 10–35 years, with a slight prevalence in Non-Hispanic white people [3]. IBD has been grossly divided into two subtypes—ulcerative colitis (UC) and Crohn’s disease (CD). While the disease presents similar clinical features in both subtypes, there are some differing characteristics which are essential for proper treatment. Yangyang and Rodriguez have listed the following symptoms: diarrhea, constipation, pain or rectal bleeding, bowel movement urgency, tenesmus, abdominal cramping, and nausea or vomiting [4]. However, there are some key differences as distinguished by the CDC. Specifically, CD can affect any part of the gastro-intestinal (GI) tract, but most often affects the small intestine. The damaged areas appear in patches and inflammatory process may reach through the layers of the walls of the GI tract. On the other hand, UC occurs in the large intestine with continuous damaged areas, usually beginning at the rectum and spreading further into the colon. This inflammation only presents on the innermost layer of the lining of the colon [5]. Gut microbial changes may also be seen in chronic IBD such as disrupted microbial composition and systemic biochemical abnormalities. Crypt abscesses are seen in UC, while CD usually has a “cobblestone” appearance in endoscopic view. 

Many scientists have been on the search for the cause of this disease; however, no such cause has been identified. All that is known is that it is the result of an improper immune system, and that genetics also plays a key role in the development of this disease. The World Journal of Gastroenterology has stated that “although the etiology of IBD remains largely unknown, it involves a complex interaction between the genetic, environmental or microbial factors and the immune responses” [2]. While still being rather uneducated about the causes of this somewhat debilitating disease, some treatment options have proved to be rather effective, with their goal being to achieve clinical and endoscopic remission. Many immunosuppressive drugs have proven to be efficient in treating IBD symptoms [6]. These include steroids, immunomodulators (thiopurines, methotrexate) and biologics such as anti-tumor necrosis factor (anti-TNF) alpha agents 4 integrins and anti-IL-12/23 agents [6]. Notwithstanding the competence of these treatments, they should be personalized for each patient. Personalization of treatment is done testing biomarkers such as calprotectin (Cal), serum C-reactive protein (CRP), fecal lactoferrin (Lf) and polymorphonuclear neutrophil elastase (PMN-e), especially used to differentiate IBD patents from those with irritable bowel syndrome (IBS) [7]. There have been some diets outlined that have shown some results in the IBD community, such as the Specific Carbohydrate Diet (SCD), the low fermentable oligosaccharide, disaccharide, monosaccharide, and polyol (FODMAP) diet, the Paleolithic diet (Paleo), and the anti-inflammatory diet (IBD-AID) [8]. Even though these have shown some improvement in IBD symptoms and management, some specific substances should be analyzed for their benefits or drawbacks in the course of IBD. Such substances are probiotics, polyphenols, fibers, fatty acids, as well as certain low FODMAP diets to be discussed. 

## 2. The Impact of Probiotics on IBD Pathogenesis

Probiotics are a very common topic of discussion among today’s health professionals. Often, they are prescribed at the same time as antibiotics to ensure replacement of intestinal microflora after ingesting somewhat harmful, but necessary antibiotics. However, there has been some recent interest in probiotics for the improvement of IBD symptoms and to alter the course of the disease. Probiotics are, generally, defined as food ingredients made from live bacteria that, when ingested in proper amounts, they change the microflora and lead to a health benefit for the host. IBD patients suffer from a loss of intestinal flora which may affect the course of the disease. “Several studies have found an imbalance in the gut microbiota in IBD patients compared to non-IBD controls with an overall loss of diversity, a depletion of firmicutes and an increase of Proteobacteria” [9,10]. These results suggest a correlation between probiotics and the course of the disease. However, these results do not seem consistent with all types of gut microbiota. Curiously enough, there was a surprising result observed. Although there was an increase of *Bifidobacterium* and the *Lactobacillus* group in active CD and UC patients, these findings did not significantly differ from the healthy group. Scientists also discovered that levels of *Clostridium coccoides* group were drastically decreased in the feces of both active UC ad CD patients. This bacterial group has been reported to affect its host in several ways. Specifically, it produces butyrate which contributes to inflammation prevention, as well colonic motility. *Clostridium coccoides* has also been noted to induce regulatory T-cell production in mice colons suggesting that these bacteria play critical roles in immune homeostasis [11]. *Escherichia coli* was also found in abundance in both types of IBD patients, yet, a higher proportion in active UC patients [12]. *Escherichia coli* presence in the intestine is known to promote intestinal homeostasis and is used as a preventative measure against pathogen colonization [13].

Recently, Dore et al. published a cohort study reporting that there was no need for systemic steroids, hospitalization and surgery in those UC patients that were taking probiotics more than 75% of the duration of the disease and there was a decrease of 93% noted in CD patients [14]. Probiotics consumed were the following: VSL#3 (450 billion CFU/packet comprising strains of *Lactobacilli*, *Bifidobacteria*, and *Streptococcus*) once daily, *Lactobacillus reuteri* (DSM 17,938) 10^8^ CFU/tablet once a day, and a mixture of *Streptococcus thermophilus*, *Lactobacillus acidophilus*, *Bifidobacterium breve*, and *Bifidobacterium animalis ssp. lactis* for a total of 50 × 10^9^ CFU/packet once a day. Matthes et al. studied 90 patients in 2010 using *Escherichia coli* Nissile 1917 in enema form for at least 2 weeks [15]. Patients had their UC disease activity index tested afterwards to determine whether the disease showed remission or not. Tolerance was observed in 80 patients proving that this may be an efficient alternative for UC treatments. In 2010, Tursi et al. studied the effects of VSL#3, a mixture of 4 strains of *Lactobacilli*, 3 strains of *Bifidobacteria* and one strain of *Streptococcus thermophilus* on 144 patients with UC [16]. 71 patients in the control group were given 2 sachets containing the above-mentioned bacteria (VSL#3) twice daily for a duration of 8 weeks. After the treatment, patients were asked about clinical remission using the UC disease activity index. There was a decrease in disease activity in those patients treated with VSL compared to the placebo group (*p* = 0.010). The researchers also noted that VSL#3 reduced rectal bleeding and significantly reduced the UCDAI scores compared to placebo. VSL#3 efficacy was also tested in CD patients in 2015 by a study conducted by Fedorak [17]. This study included 120 participants receiving 1 packet of VSL#3 daily for 3 months’ time. Afterwards, patients underwent endoscopic testing to record any changes in colonic mucosa. Doctors did not record any changes in endoscopy between the control and placebo group; however, the control group had lower mucosal levels of inflammatory cytokines and a lower rate of recurrence compared to the placebo group. This suggests that further investigation is needed for the possible effects of VSL#3 on CD disease remission. Marteau et al. studied the effects of *Lactobacillus johnsonii* in 98 patients with CD having undergone recent intestinal resection [18]. The control group was given 1 packet (2 × 10^9^ cfu per packet) of *Lactobacillus johnsonii* bacteria daily for 6 months, after which, the patients were tested endoscopically for recurrence. The study concluded that there was not a sufficient effect on the recurrence of CD. 

These studies prove that there is a relationship between bacterial species in probiotics and IBD disease as seen in Table 1. Nonetheless, there is not enough data to conclude whether or not this interaction is beneficial and, if so, to what degree. However, studies have shown the relationship between probiotics and mucosal immune systems. This relationship is mediated by Toll-like receptors to promote T-helper 1 cell differentiation, so increasing antibody production and phagocytic and natural killer cell activity, which leads to induced T-cell apoptosis and up-regulating anti-inflammatory cytokines, thus reducing inflammation in the gut, which alleviated IBD symptoms [17].

The studies have shown, however, that there is some difference between various population groups and IBD patients, suggesting that if there were some favorable medical treatments, they would need to be individual to each patient’s needs as opposed to a general therapy option. 

## 3. The Impact of Polyphenols on IBD Pathogenesis

Polyphenols are natural occurring nutrients found in plant-based substances. They are largely found in fruit, vegetables, cereal, coffee and red wine [20]. They act as antioxidants, preserving cells and body chemicals against any damage that may be caused by free radicals, reactive atoms that may cause harm to the tissues in the body. They are considered a protective method for the body. Polyphenols are known to be the most abundant antioxidant in the human diet, they are consumed, “10 times more than vitamin C and 100 times more than vitamin E and carotenoids” [21]. Polyphenols are ingested by an average of 900 mg/day [22]. Most vigorous testing and health benefit studies did not start until the mid-1990s; however, it has sparked many scientists interest in the pursuit of the polyphenols, potential, protective roles. This is speculated to be due to their wide variety and structure. Nonetheless, a study in 2017, has investigated the health effects of polyphenols in mice with IBD. Scarano et al. specifically studied the effects of flavanols, stilbenoids and anthocyanins in mice and their effect on inflammatory processes seen in IBD [23]. IBD disorders are characterized with excessive and uncontrolled response of the immune system in the intestinal mucosa against natural body microflora. Polyphenols have been studied as a preventative method in IBD patients by modulating intestinal flora [24]. This is partially due to the antioxidant effects of polyphenols, but also due to their anti-aging, anti-apoptotic, and anti-inflammatory effects. These anti-inflammatory effects were also found in in vitro studies. “Most polyphenols had an anti-inflammatory behavior by inhibiting the activation of the NF-kB cascade at, at least, one of the following steps: inhibitor of NF- kB kinase (IKK) enzymatic activity controlling the inhibitor of kB (IkB) phosphorylation, IkB phosphorylation/ degradation associated with the active NF-kB release and NF-kB transcriptional activity itself” [24]. The NF-kB pathway has many important functions, but mainly it plays a vital role in inflammatory responses at the cellular level. 

Some studies have been performed to study the effects of pure polyphenol standards on intestinal inflammation in both in vivo and in vitro studies. It is important to point out that many of the positive effects of polyphenols are observed in much higher levels than those that humans are naturally exposed to through diet. Nonetheless, if continued research proves to be as efficient as what is seen to date, increased polyphenol intake could be used as a successful treatment in CD and UC. A study published in 2019 has observed therapeutic properties of *Thuja occidentalis* when administered in medium to high doses, which was shown to inhibit inflammatory process induced by TNBS (trinitrobenzenesulphonic acid) in the intestine [25]. The scientists conducting this study concluded that the most likely explanation is the high number of flavonoids and phenolic compounds found in the substance. In 2014 a study was conducted on rats, testing the effectiveness of quercetin in rats with acetic acid induced IBD-like symptoms. Quercetin, a flavanol found in many fruit and vegetables, is the most studied polyphenol in intestinal inflammation [24]. Dodda, Chhajed and Mishra noted that there was significant improvement in ulcers in the rats, and concluded that quercetin has shown to provide some protective role in IBD-like symptoms. The mechanism behind this positive response is that quercetin affects the oxidative stress which enables the reversal of local inflammatory response and, in turn, causes hemorrhage lesion healing [26].

Curcumin, a compound derived from the rhizome of *Curcuma longa* is a type of curcuminoid. It is responsible for the yellowing coloring of many dishes and is widely studied for its anti-inflammatory effects—in this case, studied for its potential use in patients with IBD. Hanai et al., studied 89 patients with inactive UC. Forty-five patients received 1 gram of curcumin after breakfast and 1 gram after dinner along with mesalamine, and 44 other patients received placebo with mesalamine or sulfasalazine. Main results showed that 4.65% relapsed on curcumin, while 20.51% relapsed in the placebo group [27]. In 2014, Singla et al. studied the effects of curcumin enemas and placebo enemas on 45 patients with mild to moderate distal UC. There was a higher improvement in the disease activity noted in patients with curcumin enema [27]. Lang et al. looked at the effects of 5 ASA (5-aminosalicylate) administered with curcumin capsules (3g/day) or placebo with 5 ASA. 54% of patients receiving curcumin achieved clinical remission, unlike those in the placebo group [28]. Lastly, Kedia et al. studied 41 patients on a low dose of curcumin (150mg) for 8 weeks added to mesalamine. This was not effective in inducing clinical remission or response in patients suggesting that a higher dose might be necessary [29].

Resveratrol is another known naturally occurring polyphenol. It is detected in many plants and can be found in the skin of grapes, blueberries, and peanuts. Many studies have demonstrated that resveratrol has high antioxidant, as well as, anti-inflammatory potential [30]. Samsamikor et al. studied 56 patients and the effects of resveratrol on UC. Part of this group received 500 mg of resveratrol per day, while the other received a placebo dose. The group receiving resveratrol showed decreased serum level of malondialdehyde (MDA) which correlates with lower levels of oxidative stress and a significant decrease of disease activity with improved quality of life (*p* ≤ 0.001) [31]. 

The studies summarized in Table 2 have shown some link between polyphenols and UC/CD. There is abundant evidence suggesting that there needs to be more research into the use of polyphenols, specifically quercetin, in the treatment of some IBD symptoms, as well, as its possible protective roles for the gut microbiota. Curcumin and resveratrol have also shown some promising results that may be beneficial for patients suffering from IBD by decreasing disease activity and achieving clinical remission. 

## 4. The Impact of Fiber on IBD Pathogenesis

The fate of IBD patients is somewhat problematic as there is no cure for the disease to this date. Therapeutic options have shown some success in clinical settings and have induced disease remission; however, it is not a curative treatment. Therefore, research into new methods is necessary to potentially find some other effective ways to alleviate symptoms in these patients. Fiber is one of these substances that has sparked some recent interest within the scientific community, hypothesizing that the effect may be due to direct interactions with gut mucosa or indirectly through the microbiome [33].

The Codex Alimentarius Commission has defined fibers as “carbohydrate polymers with ten or more monomeric units, which are not hydrolyzed by the endogenous enzymes in the small intestines of humans and belong to subsequent categories” [34]. The influence of fibers in the etiology of CD and UC are poorly understood. Low residue diets may be recommended in those patients with active forms of disease, but in inactive patients, dietary fiber is seldom considered due to individual differences in dietary tolerances [35]. Health effects vary among the various types of fibers, but may include reducing diarrhea/constipation, producing short-chain fatty acids, down-regulating inflammation, and promoting tissue healing. These effects all play a role in the prevention of colorectal cancer onset in susceptible IBD patients [36]. Colonoscopy and gastroscopy have proved to be the most efficient tools to assess the extent of the disease and its activity in IBD [37]. Along with these imaging methods, clinicians have used visual analog scales and scores, such as CDAI (Crohn’s Disease Activity index) and Harvey-Bradshaw index, to assess effectiveness of therapy. Biomarkers, such as CRP, ESR from serum, fecal calprotectin and IL-10 are also used to determine the extent of changes [38,39].

Many types of fibers, such as fructans, psyllium, oat bran, and barley foodstuff, have been studied for their effects on IBD patients. Several studies looking into the effects of fructans were compared. A study conducted in 2011 involved Chicory fructan supplementation as Synergy 1© product 15 g/day in a randomized controlled trial, double-blind for 4 weeks for patients with active CD. There were 103 patients in total, 54 of those received fructan supplementation, while the rest had a placebo with maltodextrin. The results of this study showed decreased disease activity, increased fecal bifidobacterial counts as well as dendritic cell response [38]. The measurements of these results were obtained through various methods, such as CDAI scale, serum CRP, ESR, and fecal calprotectin measurements. The next study conducted on 19 UC patients, also using fructan in Synergy 1©, administered in the dose of 4 grams, 3 times a day for 14 days in a pilot randomized control trial. Of these 19 patients, 9 were given placebo medication. The following outcomes were observed: a decrease in dyspeptic symptoms and a decrease in calprotectin on day 7, thus indicating improvement in disease activity [40]. In short, the studies conducted to date imply that the addition of fructans into the diet may relieve GI symptoms, as well as increase gut immune function, reduce inflammation and positively modulate GI microbiota.

Psyllium has shown some benefit in improving gastrointestinal symptoms in a study conducted by Fernandez-Banares et al. in 1999. The study group comprised of 102 individuals, 35 of which received psyllium only, 37 received only mesalamine and the remaining 30 received both methods of treatment [41]. After a year, these patients were re-evaluated, and the findings suggested that psyllium might have comparable remission effects as mesalamine. Mesalamine is commonly used to treat IBD patients by reducing production of prostaglandins which decreases inflammation in the colon and decreases symptoms associated with UC. Hallert et al. found similar results in a study conducted in 1991, concluding that psyllium has improved gastrointestinal symptoms, such as abdominal pain, diarrhea, bloating, incomplete evacuation, and constipation in these patients. This study group was made up of 29 people, in which 16 were treated with psyllium, the others were given a placebo with crushed crispbread [42].

Hallert et al. conducted a study in 2003 on 32 patients with inactive UC that were given 60 g of oat bran daily for 12 weeks. The researchers observed an increase of 30% of butyrate in feces in week 4 which implies increased restoration of intestinal homeostasis and gut barrier function [43,44]. Another trial by Bo Liu et al. in 2015 studied the effects of oat beta-glucans (βG) which is present in high concentrations in oat bran. The study was performed on 80 mice, in which 60 mice had colitis induced using dextran sulfate sodium (DSS). Twenty mice were not induced with DSS, these, being the control group. The next 20 were given DSS only. The third group of 20 mice were given DSS and low dose of βG (500 mg/kg) and the remaining 20 were given DSS and a high dose of βG (1000 mg/kg) [45]. The observed findings showed that clinical symptoms, such as less weight loss, diarrhea and colon shortening was significantly reduced in mice that were given βG. 

A study in 2014 conducted by Faghfoori tested the effects of barley foodstuff supplementation in patients with inactive UC through a randomized control group study with a duration of 2 months. This supplementation resulted in a decreased mean serum level of CRP, and symptom improvements, such as reducing abdominal pain and cramping [46]. In 2002, Kanauchi et al. studied the effects of germinated barley foodstuff and observed a decrease in clinical activity index scores and an increase in *Bifidobacterium* concentrations in feces which have been found to aid intestinal microbial homeostasis [47]. This study was conducted on 18 patients, 11 receiving the drug. 

The above-mentioned fibers seen in Table 3 have shown a positive effect on the symptoms of IBD patients. Substances such as fructans, psyllium, oat bran, and barley foodstuff have decreased the gastrointestinal symptoms in tested patients, alleviating discomfort, and improving quality of life. The results of these studies have also demonstrated a decrease in disease activity and its progression. Continued research is necessary to reach a consensus about the ideal dosing of these substances to achieve optimal results and to potentially consider this addition to treatment guidelines in the future. 

## 5. The Impact of Fatty Acids on IBD Pathogenesis

Omega 3 and Omega 6 fatty acids are very important substances in human diet; however, there seems to be some discrepancy between their consumption. Linoleic acid has been consumed in higher amounts since the last century, which has been tied to some increase in the prevalence of CD and UC. On the other hand, higher amounts of docosahexaenoic acid (DHA) and eicosapentaenoic acid (EPA) is considered helpful. They have shown to replace arachidonic acid and inhibit pro-inflammatory mediator production, being indispensable to resolving inflammation processes. These acids are directly related to improved blood lipid levels and attenuation of inflammation processes seen in inflammatory diseases. The anti-inflammatory actions may be associates with their ability or change cell composition of the cell membrane, which in turn, alleviates symptoms and discomfort related to IBD [48].

In 2018, Scaioli et al., a study was conducted as a randomized controlled trial, involving 60 UC patients above 18 years of age. Patients undergoing this study had a Mayo score/Disease Activity Index (DAI) score more than 2 and fecal calprotectin above/equal to 150 μg/g. They were in continuous therapy for 3 months or more. This randomized controlled trial, had the patients divided into two groups: one with EPA-FFA (eicosapentaenoic acid as free fatty acid) of 500mg 2x/day and the other placebo lasting for 6 months. Clinical remission was noted at 6 months after commencing therapy in 76% of the EPA-FFA group compared to the 50% in the placebo group (*p* = 035). Calprotectin levels were also significantly reduced (63% in the treated group, *p* < 001) [49]. Prossomariti et al. tested the same supplementation and had similar results [50]. A cohort study published in 2014, reported a significant relationship between DHA intake and the development of CD. Chan et al. reported a decrease in new CD cases, equivalent to 4 individuals/100,000 inhabitants/year, exhibiting a positive correlation [51]. A double-blind, placebo-controlled, randomized trial conducted by Bassaganya-Riera et al. demonstrated that the use of 6 grams/day for 12 weeks of CLA (conjugated linoleic acid) reduced the production of TNF-α, IFN-γ, IL-17, lymph proliferation and significant drop in CDAI at week 12 (*p* = 0.013) [52]. These reductions suggest the down-regulation of pro-inflammatory cytokines leading to lower CDAI score and progress towards remission. 

In 2005, Seidner et al., conducted a double-blind placebo-controlled study on 86 patients with mild to moderate UC. Patients were divided into a control group (50 patients) which received a liquid supplementation based on sucrose and the active substance group (36 patients) which received 2.5 g of EPA and 1.0 g of DHA per day for 6 months. Since both groups were suffering from active UC, they were also taking corticosteroids symptomatically. The study resulted in no significant difference between both groups; however, the group receiving EPA and DHA were less likely to take steroids, and did not exceed baseline dose during the study (*p* < 0.001) [53]. A study by Feagan et al., in 2008, tested 365 adult patients in remission of inactive CD, treating them with enteric coated capsules with 2–2.4 g of EPA and 0.6-1 g of DHA per day. The placebo group received MCT (medium-chain triglyceride oil). Patients were not permitted to use any other medications. The results showed that the relapse rate was lower (36.1% relapse rate), than the placebo group (35.7% relapse rate) (*p* = 0.30) [54].

In general, the fatty acids in question, listed in Table 4, have shown to be associated with clinical remission and decreased disease activity. They have shown to help reduce the use of corticosteroids throughout the duration of the disease, which has long-term benefits. The use of fatty acid additives in diet could prove to be an effective complementary treatment to current therapies. Although more studies need to be conducted to evaluate the most effective dosage and long-term effectiveness, the studies suggest a potential, viable alternative or additive to current medication. 

## 6. The Impact of Low FODMAP Diet on IBD Pathogenesis

As IBD has become a more prevalent problem in the Western world, many scientists have been studying various diets to potentially find a way to ease patient’s lifestyle and progression of disease. Many substances have been advised against by physicians worldwide, such as coffee, spicy foods, alcohols as they have been noted to worsen patient’s condition and decrease comfort. With this being said, there are some substances that have been noted to ameliorate the quality of life in IBD patients by reducing their IBS-like symptoms. It is estimated that more than 30% of patients with IBD have concomitant IBS and that functional gastrointestinal symptoms are observed approximately 3 times more frequently in patients with IBD compared to the general population [55,56]. Specific diets have been put together and may be recommended to these patients, including beneficial substances. Such a popular diet is known as a low fermentable oligo-, di-, mono-saccharide and polyol diet (low FODMAP diet).

Low FODMAP diet is a dietary restriction of fermentable oligosaccharides, disaccharides, monosaccharides, and polyols, referred to as “FODMAPs”. These are short-chain, highly fermentable and poorly absorbed carbohydrates that lead to intestinal dysbiosis, injury/inflammation, excess gas formation and luminal distension through bacterial fermentation, and water secretion [57]. The following foods are recommended when following a low FODMAP diet: meat, poultry, fish, eggs, lactose-free dairy, gluten-free grains (i.e., rice), fruit (i.e., bananas), vegetables (i.e., spinach), most nuts, most spices and herbs. Avoiding foods that have high lactose content, legumes, garlic, and onion. Patients are recommended to follow strict dietary restrictions for the first 4–6 weeks before introducing some FODMAPs and closely monitoring any change in symptoms to arrive at an optimal level of FODMAP restrictions to best suit their individual needs [58] A study published in 2017 by Pederson et al. studied the effects of a low FODMAP diet on 78 adult patients with IBD and IBS-like symptoms. After 6 weeks, the low FODMAP diet group showed a significant reduction in IBS symptoms and a higher score in a questionnaire regarding their quality of life (*p* < 0.01) [59]. Cox et al., conducted another study in 2017 on 32 patients with IBD who also had functional gastrointestinal symptoms, functional bloating, or functional diarrhea. Patients were randomly divided into groups receiving a carbohydrate (fructan, galacto-oligosaccharides (GOS) or sorbitol) or the placebo group receiving glucose. The participants had to drink a challenge drink once daily for 3 days followed by a “washout” period lasting 4 days minimum. They would then be required to drink one of the other substances in the same protocol until all substances were consumed. Gastrointestinal symptoms and stool output were recorded before each challenge was begun and right after it was completed. Once all four challenges and the final washout period were completed, the noted symptoms were analyzed and these are the results: fewer patients reported amelioration of gastrointestinal symptoms (62.1%), compared to the group consuming glucose (89.7%) (*p* = 0.033). There was also a greater severity of pain (*p* = 0.004), bloating (*p* = 0.002), flatulence (*p* = 0.004) and fecal urgency (*p* = 0.014) on the final day of the fructan challenge compared to glucose [60]. Similarly, Prince et al. found a significant increase in numbers of patients reporting satisfactory relief of symptoms with the implementation of a low FODMAP diet. (*p* < 0.001) [61].

The evidence regarding the effectiveness of a low FODMAP diet is compelling, showing a positive correlation between low FODMAP and reduction in clinical gastrointestinal symptoms and discomfort, as can be seen in Table 5. This, of course, will lead to an increased quality of life. It is important to note as well that as inflammation and intestinal injury occurrences decrease, the long-term effects are less dramatic and life altering. Although the studies show a positive effect, they also conclude that this diet is very specific to each individual, as each patient can tolerate different amounts of FODMAPs and thus, have different symptom relief. The guidance of a clinical dietician would prove to be beneficial and would help patients reach an optimal level which would best fit their everyday needs. 

## 7. Conclusions

Most substances reviewed showed a positive effect on patients with IBD and their clinical symptoms. Fibers have also shown similar results. Those fibers, such as fructans, psyllium, oat bran, and barley foodstuff have relieved some gastrointestinal symptoms that have been associated with IBD, this has improved patient’s quality of life. The fatty acids reviewed have proved to be effective in decreasing corticosteroid use among may IBD patients. Probiotics have shown some promising results; however, more testing is required to provide definite results. Lastly, low FODMAP diets have also shown a very positive effect in decreasing uncomfortable gastrointestinal symptoms. 

Many patients reached some remission, and those that did not, did not have any worsening progression of disease. This suggests that in the worst-case scenario, there is no negative effect after implementing these substances into the diet. However, that being said, there is a need for further studies to gauge the right amount of each substance to generate the highest yielding results. Implementing fibers, polyphenols, and fatty acids, as well as keeping a low-saccharide diet for those patients with CD and UC can improve quality of life and invoke clinical remission. Some polyphenols, specifically curcumin and resveratrol, have proved to decrease disease activity in studies reviewed. This may also be a cheaper option to some of the current therapies on the market, or may also be used in conjunction with therapies to help with symptoms and clinical remission. Regardless of the substance, diets and implementation of these substances should be done with the guidance of a dietician or doctor familiar with the effects. This will also allow for the proper dosage to achieve optimal treatment results. 

## Figures and Tables

**Table 1:** Summary of trials examined for effectiveness of probiotics on IBD.

Trial Author	Number of Patients	Treatment	Duration	Results/*p* Value
Dore et al., 2018 [14]	200 patients (78 with CD, 122 with UC)	Probiotics given orally; VSL#3 (450 billion CFU/packet comprising strains of *Lactobacilli*, *Bifidobacteria*, and *Streptococcus*) once daily; *Lactobacillus reuteri* (DSM 17,938) 108 CFU/tablet once a day; and a mixture of *Streptococcus. thermophilus*, *Lactobacillus acidophilus*, *Bifidobacterium breve*, and *Bifidobacterium animalis* ssp. *lactis* for a total of 50 × 109 CFU/packet once a day	36 months	CD patients taking probiotics for 25–74% of disease, had a 64% reduction. Systemic steroid use and hospitalizations dropped to zero events for UC patients and decreased by 93% (*p* < 0.001) in CD patients taking probiotics for ≥75% of the disease duration.
Matthes et al., 2010 [19]	90 patients with UC	*Escherichia coli* Nissile 1917 enema	2 weeks	80 patients had positive effects.
Tursi et al., 2010 [15]	144 patients with UC	Mixture of 4 strains of *Lactobacilli*, 3 strains of *Bifidobacteria* and one strain of *Streptococcus thermophilus* (VSL#3) taken orally	8 weeks	Decrease in disease activity compared to the placebo group (*p* = 0.010).
Fedorak et al., 2015 [16]	120 patients with CD	1 packet of VSL #3 daily, taken orally	3 months	Control group had lowered mucosal levels of inflammatory cytokines and lower rate of recurrence.
Marteau et al., 2006 [20]	98 patients with CD with recent intestinal resection	1 packet *Lactobacillus johnsonii* bacteria daily taken orally	6 months	Concluded that there was not any sufficient effect.

**Table 2:** Summary of trials examined for effectiveness of Polyphenols on IBD.

Trial Author	Number of Patients	Substance Tested	Duration	Results/*p*-Value
Stan et al., 2019 [25]	100 male mice	*Thuja occidentalis* 25 or 50 mg/kg/per day	7 days	Shown to inhibit inflammatory processes induced by TNBS.
Dodda et al., 2014 [26]	6 rats	Quercetin 50 mg/kg/per day or 100 mg/kg/per day.	3 days	Significant improvement in ulcers in rats, and shown some protective role in IBD symptoms.
Hanai et al., 2006 [32]	89 patients with inactive UC	1 gram of curcumin after breakfast and 1 gram after dinner along with mesalamine.	6 months	4.65% relapsed on curcumin (*p* = 0.40).
Singla et al., 2014 [27]	45 patients with mild to moderate UC	Curcumin NCB-02 enemas containing 140 mg of NCB-02 (curcumin) preparation dissolved in 20 mL of water.	8 weeks	Higher improvements in disease activity noted in patients receiving the enema (*p* = 0.14).
Lang et al., 2015 [28]	50 patients with UC treated with mesalamine	5-aminosalicylate administered with curcumin capsules.	1 month	Curcumin addition helped to achieve clinical remission (*p* ≤ 001).
Kedia et al., 2017 [29]	41 patients with mild to moderate UC	150 mg of curcumin added to 2.4 g of mesalamine	8 weeks	Not effective in inducing clinical remission or response in patients tested (*p* = 0.75).
Samsamikor et al., 2016 [31]	56 patients with UC	500 mg of resveratrol	6 weeks	Decreased serum level of malondialdehyde (MDA), which decrease disease activity (*p* = 0.001).

**Table 3:** Summary of trials examined for effectiveness of Fibers on IBD.

Trial Author	Number of Patients	Substance Tested	Duration	Results/*p*-Value
Benjamin et al., 2011 [38]	103 patients with active CD	Chicory fructan supplementation Synergy 1 © product 15 g/day	4 weeks	Decreased disease activity, increased fecal bifidobacterial counts, and dendritic cell response (*p <* 0.05).
Casellas et al., 2007 [40]	19 patients with UC	Synergy 1©, administered in the dose of 4 grams, 3 times daily	14 days	Decrease in dyspeptic symptoms, decrease in calprotectin on day 7 (*p <* 0.05).
Fernandez-Banares et al., 1999 [41]	102 patients with UC	Psyllium 10 g, twice daily	1 year	Comparable results of remission as mesalamine (*p* = 0.67).
Hallert et al., 1991 [42]	29 people with UC	Psyllium (3.52 g ispaghula husk)	4 months	Improved gastrointestinal symptoms (*p <* 0.001).
Hallert et al., 2003 [43]	32 patients with inactive UC	60 g of oat bran daily	12 weeks	Increased restoration of intestinal homeostasis and gut barrier function (*p <* 0.05).
Faghfoori et al., 2014 [46]	46 patients with inactive UC	Barley foodstuff supplementation 30 grams, 3 times daily)	2 months	Decreased CRP, and symptoms improvements (*p* = 0.017).
Kanauchi et al., 2002 [47]	18 patients with moderate UC	Germinated barley foodstuff (20–30 grams daily)	4 weeks	Decrease in clinical disease activity and increase in *Bifidobacterium* numbers in feces, aiding intestinal microbial homeostasis (*p <* 0.05).
Liu et al., 2015 [45]	80 mice	Oat beta-glucans, 500 mg/kg /day or 1000 mg/kg/day	7 days	Clinical symptoms such as weight loss, diarrhea and colon shortening significantly reduced (*p <* 0.05).

**Table 4:** Summary of trials examined for effectiveness of Fatty Acids on IBD.

Trial Author	Number of Patients	Substance Tested	Duration	Results/*p* Value
Scaioli et al., 2018 [49]	60 UC patients	EPA–FFA (eicosapentaenoic acid as free fatty acid), of 500 mg 2×/day	6 months	Clinical remission was noted in 50% compared to placebo (*p* = 035).
Prossomariti et al., 2017 [50]	20 patients with UC	EPA–FFa 2 g/daily	3 months	Reduced mucosal inflammation, promoted goblet cell differentiation, and modulated intestinal microbiota.
Chan et al., 2014 [51]	229, 705 patients across 9 European centers	DHA intake and risk of CD	7 years	A significant relationship between DHA intake and the development of CD was noted.
Bassaganya-Riera et al., 2012 [52]	13 patients with mild to moderate CD	6 g/day of CLA (conjugated linoleic acid)	12 weeks	Reduced the production of TNF-α, IFN-γ, IL-17, lymph proliferation and significant drop in CDAI at week 12 (*p* = 0.013).
Seidner et al., 2005 [53]	50 patients	2.5 g of EPA and 1.0 g of DHA per day	6 months	EPA and DHA group were less likely to take steroids and did not exceed baseline dose (*p <* 0.001).
Feagan et al., 2008 [54]	365 adult patients in remission of active CD	Enteric coated capsules with 2–2.4 g of EPA and 0.6–1 g of DHA per day	4 years	Relapse rate was lower (36.1%) in control group (*p* = 0.30).

**Table 5:** Summary of trials examined for effectiveness of Low FODMAP diet on IBD.

Trial Author	Number of Patients	Substance Tested	Duration	Results/*p*-Value
Pederson et al., 2017 [59]	78 patients with IBD and IBS-like symptoms	Low FODMAP diet	6 weeks	Significant reduction in IBS symptoms (*p <* 0.01).
Cox et al., 2017 [60]	32 patients with IBD	Carbohydrates	~4 weeks	Greater severity of pain (*p* = 0.004), bloating (*p* = 0.002), flatulence (*p* = 0.004), and fecal urgency (*p* = 0.014) were noted.
Prince et al., 2016 [61]	88 patients with IBD	Low FODMAP diet	6 weeks	Significant and large increase in relief of baseline symptoms (*p* < 0.001).
